# Zoonotic Soil-Transmitted Helminths in Free-Roaming Dogs, Kiribati

**DOI:** 10.3201/eid2708.204900

**Published:** 2021-08

**Authors:** Patsy A. Zendejas-Heredia, Allison Crawley, Helen Byrnes, Rebecca J. Traub, Vito Colella

**Affiliations:** University of Melbourne, Parkville, Victoria, Australia (P. Zendejas-Heredia, R.J. Traub, V. Colella);; independent researcher, Brisbane, Queensland, Australia (A. Crawley); Vets Beyond Borders, Brisbane (H. Byrnes)

**Keywords:** zoonoses, helminths, hookworms, *Strongyloides*, dogs, prevention, Kiribati, parasites

## Abstract

Soil-transmitted helminths are highly prevalent in the Asia–Pacific region. We report a 96.5% prevalence of zoonotic soil-transmitted helminths in dogs in Kiribati. We advocate for urgent implementation of treatment and prevention programs for these zoonotic pathogens, in line with the Kiribati–World Health Organization Cooperation Strategy 2018–2022.

Soil-transmitted helminths (STHs) are a group of parasitic worms infecting both humans and animals living in resource-limited settings (*1*). STHs affect >2 billion persons worldwide, causing major physical and cognitive impairment in children and negative health outcomes in pregnant women and women of childbearing age (*2*). Hookworms alone infect nearly half a billion persons, causing iron-deficiency anemia, stunted growth, and malnutrition (*3*). In addition, iron deficiencies may increase the risk of bacterial infections, especially in children <5 years of age (*3*). Although STHs are largely considered human-specific parasites, dogs are also known to harbor STHs that cause well-documented zoonotic diseases globally (*3*). The *Ancylostoma ceylanicum* roundworm is a zoonotic STH with dogs as reservoirs and is the second most common hookworm infecting humans in many regions in Southeast Asia and the Western Pacific (*3*). In humans, canine hookworms cause cutaneous larva migrans; *A. braziliense* hookworm is the only species capable of causing creeping eruptions and *A. caninum* hookworm triggers eosinophilic enteritis and aphthous ileitis (*4*). Recently, *A. caninum* eggs have been reported in the feces of human patients, suggesting that this parasite may complete its life cycle in humans, which can potentially result in disease transmission between hosts (*4*). In dogs, infections with hookworms are a common cause of hemorrhagic diarrhea and death in pups and chronic iron deficiency anemia in adult animals (*4*). The existence of dogs in close proximity to humans living in poor-hygiene settings, coupled with a lack of veterinary services and zoonotic awareness, exacerbates infection risks for the transmission of zoonotic STHs (*5*). In the Pacific islands, data on STH prevalence is scarce; therefore, estimates of disease burden caused by STHs cannot be accurately assessed. 

Kiribati is a sovereign state in Micronesia in the central Pacific Ocean and is one of the most geographically isolated and impoverished countries in the world (*6*). Effects of poverty and climate change exert a huge toll on the ecology and health of humans and animals inhabiting the country. For instance, in the capital, South Tarawa, the high level of poverty, overcrowding, and presence of free-roaming animals influence the epidemiology of zoonotic STHs, trapping poor persons in a vicious cycle of poverty (*6*,*7*). Despite the Kiribati–World Health Organization Cooperation Strategy 2018–2022 (*6*), to date, no information is available on the presence and diversity of zoonotic STHs in free-roaming animals in Kiribati.

## The Study

The republic of Kiribati consists of 32 atolls in the central Pacific Ocean, with a population of >110,000 persons, inhabiting mainly the Gilbert Islands. The main economic revenue comes from seafood exports and fishing. Most primary foods are imported, and safe water supplies, proper solid waste disposal, and sanitation facilities are scarce, posing major threats to public health (*6*).

**Figure 1:**
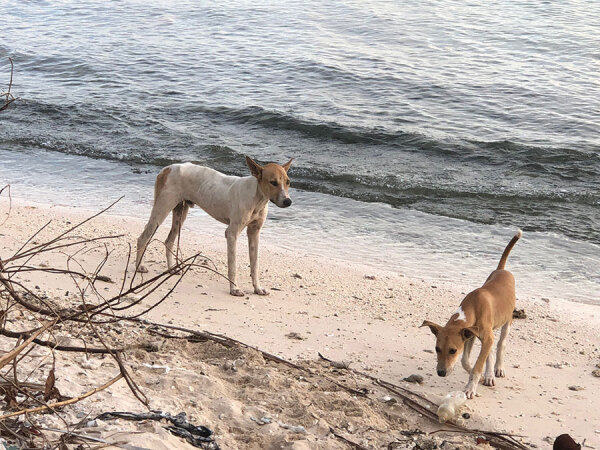
Living conditions of dogs on Tarawa Atoll, Kiribati.

We investigated the occurrence of zoonotic STHs in free-roaming dogs in Tarawa Atoll, Kiribati, as part of a dog health and population management program led by the Mardi Chi Dingo Foundation (https://farriervet.com/mardi-chi; Figure 1), which aims to seek a sustainable locally driven solution to improving animal health and overpopulation problems. The protocol of this study was approved by the Animal Ethics Committee at the Faculty of Veterinary and Agricultural Sciences (University of Melbourne, Melbourne, Victoria, Australia; ethics identification no. 1914930).

On the basis of previous surveys in regions with similar ecologic conditions (≈80% expected prevalence of enteric parasites) (*5*), we estimated that ≈200 dogs needed to be sampled in Kiribati to assess the prevalence of zoonotic STHs with 95% confidence and to detect a pathogen, if present, at a prevalence of >0.5% (assuming diagnostic sensitivity of 80% and specificity of 98%). To minimize animals’ stress, we collected fecal samples from the rectal ampulla coinciding with the dog being anesthetized just before desexing surgery. We immediately preserved the fecal samples in Zymo DNA/RNA Shield (Zymo Research, https://www.zymoresearch.com), which renders any potential pathogen inactive or noninfective. We subjected fecal samples (200 mg each) from 198 dogs to genomic DNA extraction at the University of Melbourne using a Maxwell RSC PureFood GMO and Authentication Kit (Promega, https://www.promega.com) according to the manufacturer’s instructions with modifications in that we performed an additional bead-beating step with 400 μL CTAB buffer using 0.5 mm zirconia/silica beads (Daintree Scientific, http://www.daintreescientific.com.au) using a FastPrep-24 5G Instrument (MP Biomedicals, https://www.mpbio.com). After bead-beating and cell lysis, we proceeded with DNA purification in a Maxwell RSC 48 Instrument (Promega). We stored the final eluted sample (100 μL) at −20°C for further analyses. We subjected the extracted DNA to multiplex quantitative PCR screenings for hookworm species (*4*) and *Strongyloides stercoralis* (*8*). We analyzed and visualized the data with GraphPad Prism version 8.0 (GraphPad Software, https://www.graphpad.com).

**Figure 2:**
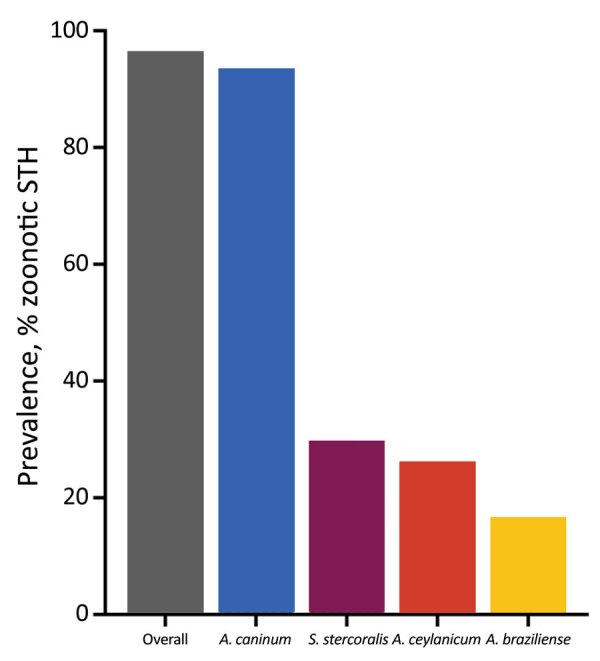
Prevalence of zoonotic soil-transmitted helminths in dogs on Tarawa Atoll, Kiribati.

Overall, 96.5% (95% CI 93.9–99.0) of dogs were positive for >1 of the investigated parasites. A total of 93.4% (95% CI 92.5–98.4) were positive for *A. caninum*, 26.3% (95% CI 20.1–32.4) for *A. ceylanicum*, 16.2% (95% CI 11.5–21.9) for *A. braziliense*, and 29.8% (95% CI 23.4–361) for *S. stercoralis* (Figure 2).

## Conclusions

We demonstrated that dogs play a major role in contaminating the environment with zoonotic STH species, potentially serving as reservoirs for infections of humans living in Kiribati. Current control strategies against STHs in Kiribati have been based on deworming of school-age children as part of the Pacific program to eliminate lymphatic filariasis using albendazole as prophylactic treatment (*9*). However, this drug has limited effects against *S. stercoralis*, which requires ivermectin for its effective control and for which public health strategies are yet to be developed (*10*,*11*). Similarly, the emerging zoonotic agent *A. ceylanicum* has been reported with high prevalence, and despite ≈100 million persons currently infected with this STH (*12*), to date, no plan exists for its control. Given the different transmission dynamics and infection outcomes with different zoonotic STHs, accurate identification of these parasites is essential for the implementation of effective therapy and control programs (*4*,*12*). However, despite the efforts of nonprofit organizations, data on the occurrence of canine STHs in Kiribati were not previously available, hindering the understanding of the contribution of dogs in the transmission of zoonotic pathogens to humans.

Previous studies have shown an association between helminth infections and higher levels of anemia among school-age children from the Pacific region (*13*). Children with helminth infections were 3.6 times more likely to be stunted in growth and 2 times more likely to be anemic (*13*). This scenario is worsened by the absence of effective sewage systems, which contributes to the environmental contamination with animal and human feces, as demonstrated by the high levels of fecal coliforms in samples extracted from groundwater throughout South Tarawa (*14*). The lack of appropriate water, sanitation, and hygiene procedures increases the risks for infection with human and animal pathogens, including STHs. As a consequence, pneumonia and diarrhea, which have both strong links to hygiene and water, are some of the leading causes of illness and death among children in Kiribati (*14*).

In summary, we report a 96.5% prevalence of zoonotic STHs in dogs in Kiribati. Our results provide policy makers and key stakeholders with epidemiologic information that can be used for control programs to improve the health and quality of life of persons (in particular, women of reproductive age and children) and animals in the country, in line with the Kiribati–World Health Organization Cooperation Strategy 2018–2022.
